# Attitudes toward Same-Sex Attraction and Behavior among Chinese University Students: Tendencies, Correlates, and Gender Differences

**DOI:** 10.3389/fpsyg.2016.01592

**Published:** 2016-10-13

**Authors:** Xinli Chi, Skyler T. Hawk

**Affiliations:** ^1^College of Psychology and Sociology, Shenzhen University, ShenzhenChina; ^2^Department of Educational Psychology, Faculty of Education, The Chinese University of Hong Kong, Hong KongChina

**Keywords:** Chinese university students, same-sex attraction and behavior, gender differences

## Abstract

The present study examined Chinese university students’ attitudes toward same-sex attraction and behavior, the socio-demographic correlates of these attitudes, and the potential gender differences in both tendencies and correlates. A total of 2,644 Chinese university students (49.7% male, mean age = 20.27 years) indicated generally negative attitudes toward same-sex attraction and behavior, with males reporting more negative attitudes than females. More years in university (i.e., higher grade levels), higher levels of maternal education, growing up in an urban area, and more frequent Internet use significantly predicted more positive attitudes. Gender significantly moderated one correlate: For female participants, a higher university grade was related to more positive attitudes; this correlation was not significant for male participants. The findings suggest valuable directions for related intervention practices for young people in China.

## Introduction

An attitude is defined as “a predisposition or a tendency to respond positively or negatively to a certain idea, object, person, or situation” (Ahmad, 2008; p. 41). The literature on attitudes toward same-sex attraction, behavior, and relationships has documented that, although acceptance is growing in many Western countries, negative attitudes remain widespread across many cultures. The minority stress theory (Meyer, 2003) suggests that many gay and lesbian individuals experience unique stressors related to their sexual orientation. These stressors include homophobic prejudice, social rejection, discrimination, and harassment (Steffens and Wagner, 2004; Winter et al., 2008; Hladik et al., 2012; Heinze and Horn, 2014). Being confronted with these stressors, in turn, is linked with negative mental health outcomes (Meyer, 2003), including depression, drug use, and suicide attempts (Sandfort et al., 2001; Boehmer, 2002; Arnarsson et al., 2015). Since well-being among gay and lesbian people is a significant public health concern, in recent decades, many scholars in Western countries have addressed attitudes related to same-sex attraction and behavior, and policy makers have aimed to reduce discrimination and contribute to a generally positive social atmosphere (Harper, 2007). However, these trends have not yet fully transferred to non-Western countries, such as China, where theoretically embedded studies on attitudes toward gay and lesbian individuals—as well as research-grounded interventions aimed at improving these attitudes—are relatively scarce.

The present study, therefore, first introduces the general attitudes toward same-sex attraction and behavior in China, presents related gender theories, and reviews prior related studies. Next, the study proposes several research aims: to explore current attitudes toward same-sex attraction and behavior, along with the socio-demographic correlates of these attitudes, among a large sample of Chinese university students, and to examine gender differences in these attitudes and their correlates. Finally, the study discusses the important findings and suggests valuable directions for related intervention practices for young people in China.

### Attitudes toward Same-Sex Attracted Individuals in China

Although Western literature has demonstrated that there are many types of sexual orientation, including gay and lesbian, bisexual, and mostly gay and lesbian (Currin et al., 2015), studies of the Chinese context have reported only limited recognition of such groups (Kwok et al., 2012). Considering the scarcity of China-based literature in this field, the current study does not subdivide the categories of sexual orientation; instead, it uses the term “same-sex attracted individuals” to refer to all individuals who have same-sex sexual desires, sexual behavior, and relationships, regardless of the degree of attraction.

According to a national cross-sectional survey, it was estimated in 2006 that as many as 50 million gay men and lesbian women lived in China, and this number may be significantly larger today (Li, 2006). It was estimated this number may be significantly larger today (Li, 2006). Despite this large number and the evident presence of gay and lesbian individuals in society, Chinese people who experience same-sex attraction struggle with overwhelming societal discrimination, family backlash, and a lack of legal protection (Sim, 2014). Traditional Chinese culture (which is rooted in Confucian philosophies) emphasizes the continuation of the family line, filial piety, and the patriarchal family structure (Hsu and Waters, 2001). Additionally, in the context of the Chinese family planning policy (i.e., prior to 2016, the One-Child Policy; from 2016 to the present, the Two-Child Policy), families and proximal social surroundings exert intense pressure on gays and lesbians to marry and have children in order to protect their families’ reputations and lineages (Liu and Choi, 2006)^1^. Prejudice and discrimination toward gay and lesbian individuals in China have been linked to high levels of mental and behavioral problems, such as depression and suicide attempts (Liu and Choi, 2006; Feng et al., 2010). These findings are similar across cultures, suggesting an important contemporary public health challenge. Currently, the Chinese government takes a passive and ambiguous stance toward same-sex relationships, which state policy describes using the “‘Four No’s’: no enquiry (bu wen), no mentioning (bu ti), no talking (bu shuo), and no response (bu li)” (Zhou, 2006); however, this neutral and passive position does not imply acceptance, and this government-level ambiguity creates challenges in the management of legal and public health issues related to gay and lesbian people (Sim, 2014).

Although many scholars have pointed to the need to examine issues of discrimination toward gay and lesbian people in China, Chinese literature on these topics is scarce. Among the few related studies conducted, some evidence suggests that a large proportion of the Chinese population continues to hold negative attitudes toward same-sex sexual desires, sexual behavior, and relationships (Liu and Choi, 2006; Jun, 2010; Kohut, 2013). According to a 2013 survey by the Pew Research Center, for example, 57% of the Chinese respondents believed that “homosexuality” was unacceptable, and only 21% indicated accepting attitudes. At the same time, there is evidence that these attitudes are currently in a state of flux. The Pew study (2013) noted that positive attitudes had increased by 4% (from 17 to 21%) between 2007 and 2013. There was also a sizable age discrepancy in attitudes, with 18- to 29-year-olds being more likely to indicate acceptance of “homosexuality” (32%) than individuals aged 30 to 49 years old (19%) or 50+ years old (15%).

Furthermore, the first legal challenge arguing for same-sex marriage was recently accepted by the Changsha Furong District People’s Court, Hunan Province, in early January 2016. Although the hearing of this case has been delayed with no official explanation, advocates for gay and lesbian rights in China view even the acceptance of this case by the courts as notable progress in the government’s acknowledgment of this issue (Lu and Hunt, 2016). In addition to legal challenges, the recent spread of mass media, especially the Internet, in China has played an important role in transmitting information related to gay and lesbian issues and providing homosexual individuals ways to connect with other homosexuals within the general public (Lin et al., 2016). The spread of media may also affect the attitudes of Chinese young people toward same-sex attraction and behavior. In sum, these current events, the apparent age gap reported by recent studies, and the effects of media signal a need for additional research to shed light on predictors of Chinese young people’s attitudes toward same-sex attraction and behavior.

### Correlates of Attitudes

The extant literature indicates various socio-demographic correlates of attitudes toward gay and lesbian individuals. These include family socioeconomic status (SES), urbanity, sex education experience, and Internet use among young people. Many studies have consistently found that young individuals with higher family SES (measured in terms of family income and parental education level) generally hold more tolerant attitudes than individuals from lower SES families (Calzo and Ward, 2009; Dunn, 2010). Similarly, people living in urban areas tend to be more tolerant than people living in rural areas (Norton and Herek, 2013). These issues related to SES and urbanity are also evident in China; while larger, richer metropolitan areas, such as Beijing and Shanghai, feature thriving and relatively openly gay and lesbian subcultures, such subcultures are far less common In the poorer and more rural areas of the country. In addition to these socio-demographic correlates, prior research has found that receiving sex education helps to decrease negative attitudes toward gay and lesbian individuals (Waterman et al., 2001). A study based on Chinese university students has demonstrated that a comprehensive sex education program promotes positive attitudes toward sexual and gender identity minorities (Chi et al., 2013). Furthermore, empirical studies have revealed that exposure to the Internet significantly predicts more positive attitudes toward gays and lesbians and have suggested that media-based intervention strategies could increase such acceptance (Newman, 2007; Lou et al., 2012).

### Gender Differences in Tendencies and Correlates of Attitudes

We refer to Butler’s (2010) view in considering gender the cultural interpretation or signification of that facticity and performance through which an individual agent acts. It is noticed that there is no gender identity behind the expressions of gender; that identity is performatively constituted by the very “expressions” that are said to be its results” (Butler, 1999, p. 34). Regarding gender studies, hegemonic masculinity is one of the most popular theories for explaining gender differences and how and why males maintain dominant social roles over females. This theory suggests that males promote a hegemonic masculinity to achieve power, status, and ascendancy through culture, institutions, persuasion, and other methods (Connell, 1993; Connell and Messerschmidt, 2005). To maintain their own advantages, males are more likely to stand out against violations of traditional gender roles and sexual patterns (i.e., sexual relationship between males and females). Accordingly, males were more likely to have antipathy toward same-sex attraction, behavior, and relationship than females were. In addition, the public generally have a perception that homosexual men are sissy (Winter et al., 2009). In this manner, it is thought that homosexual men promote femininity by embodying a feminine identity hence viewed as inferior, which may result in them being targets of discrimination.

This theory is also applicable to Chinese gender roles. The orthodox Confucian doctrine, which dominates the gender role culture in China, advocates that a woman’s behavior should be governed by “Three Obediences,” as follows: “as an unmarried girl she must obey her father and brothers; as a married woman she must obey her husband; and as a widow she must obey her adult sons” (Leung, 2003, p. 361). As a result, Chinese females have traditionally been subordinated to males during every stage of their lives. To maintain their high status and power, Chinese males promote conformity to roles that emphasize these advantages and object to violations of traditional sexual patterns (i.e., sexual relationship between males and females).

In addition to having more rigid attitudes toward same-sex attraction and behavior, according to the female plasticity perspective (Baumeister, 2000), males also have more stable attitudes than females, both across time and in different situations. Females’ flexibility may be rooted in two primary aspects: innate and genetic patterns, and social learning processes and adaptation. Regarding biological patterns, researchers has suggested more X chromosome in females and more testosterone in males may leave more opportunity for the environmental influence in females than males (Bem, 1998). Concerning social learning process and adaptation, “males are physically stronger and more aggressive than women on average, and they also tend to hold greater sociopolitical and economic power” (Baumeister, 2000, p. 349). If ideas and behaviors were to differ between females and males, males would have several advantages over the woman for achieving his goals. Greater plasticity on the part of females may be one social learning or adaptive response to social power and environment (Ridley, 1993).

Echoing Connell’ theory that male have more prejudice toward same-sex attraction, behavior and relationship, empirical studies have shown that young men generally hold significantly more negative attitudes about same-sex attraction than young women (Herek, 2002; Nagoshi et al., 2008; Teney and Subramanian, 2010; Costa and Davies, 2012). Consistent with Baumeister’s perspective, prior literature has demonstrated that males’ sexual attitudes and behavior tend to remain more stable and constant across time and contexts, whereas females’ sexual ideas and behavior tend to be influenced by socio-cultural factors (Baumeister, 2000; Chi et al., 2012, 2015). While these prior studies have provided valuable information for understanding factors of attitudes concerning same-sex attraction and behavior and of gender differences in these attitudes and correlates, most were conducted in Western countries, such as the US and Europe. Thus, it remains unknown whether the aforementioned predictors and gender differences hold true in relation to the attitudes of Chinese young people.

### The Present Study

The aim of the present study was to explore attitudes toward same-sex attraction and behavior among a large sample of Chinese university students. Specifically, three research questions were designed to achieve our aim: (1) what were beliefs regarding violations of social norms, acceptance of same sex-marriage, same-sex sexual desire, and same-sex sexual behavior among these Chinese university students? (2) What were the factors (i.e., gender, grade, family SES, urbanity, sex education experience, and Internet use) predicting these attitudes? (3) What were the gender differences in both these attitudes and correlates?

Regarding the first research question, based on previous research and traditional Chinese value, we expected Chinese university students to generally report negative attitudes toward same-sex attraction and behavior (Hypothesis 1). For the second research question, based on prior literature and hegemonic masculinity theory, we hypothesized that female students (Hypothesis 2a), students with more years of university education (Hypothesis 2b), students with higher family SES (Hypothesis 2c), students from urban areas (Hypothesis 2d), students who had previously received sex education (Hypothesis 2e), and students reporting more frequent online activity (Hypothesis 2f) would report more positive attitudes than male students, students with fewer years of university education, students with lower family SESs, students from rural areas, students with no sex education experience, and students reporting less frequent online activity. Concerning the third research question, based on female plasticity perspective, we expected to find significant gender interaction effects that would indicate that women’s attitudes are more strongly associated with the investigated correlates than those of men (Hypothesis 3). Investigating these students’ attitudes toward same-sex attraction and behavior and the socio-demographic correlates of these attitudes is critical for developing effective and feasible strategies for promoting sex education (e.g., school-based, community-based, and family-based sex education programs) for Chinese adolescents and young adults and for addressing prejudice and discrimination toward individuals experiencing same-sex attraction and behavior.

## Materials and Methods

### Participants

Data for the present study were collected in Eastern and Southwestern China in late 2011. There are more than 100 public universities in Eastern and Southwestern China, covering a wide range of disciplines such as science, social science and humanities. From the over 100 universities, nine universities were randomly selected for the recruitment of the study sample. A total of 2,750 full-time university students were invited to participate via a questionnaire; all signed a consent form before filling out the questionnaire. In total, 2,644 students (96.1%) completed the questionnaire. The sample consisted of 31.8% first-year students, 35.2% sophomores, 20.4% juniors, and 12.5% seniors. The sample included 1,315 males (49.7%) and 1,329 females (50.3%), with a mean age of 20.27 years (*SD* = 1.40). Further demographic information concerning the participants is summarized in **Table 1**.

**Table 1:** Socio-demographic characteristics of the study participants.

		*N*	%
Gender	Male	1,315	49.7
	Female	1,329	50.3
Grade	Year 1	842	31.8
	Year 2	932	35.2
	Year 3	539	20.4
	Year 4	331	12.5
Father education	Low (illiteracy or primary school)	482	18.2
	Middle (middle or high school)	1,708	64.6
	High (college or higher)	454	17.2
Mother education	Low (illiteracy or primary school)	1,017	38.5
	Middle (middle or high school)	1,376	52.0
	High (college or higher)	251	9.5
Family income	Lower than middle level	184	7.0
	Middle level	1,743	65.9
	Higher than middle level	717	27.1
Urbanity	Rural	1,618	61.2
	Urban	1,026	38.8
Sex education	No	2,061	78.0
	Yes	583	22.0
Online frequency	Every two weeks	830	31.4
	Every one week	925	35.0
	Every day	889	33.6

### Procedure

The first author tried to establish contact with the professors of the surveyed universities through email and phone. With their help and introduction, the first author gained access to the universities. The survey used the paper-and-pencil survey method, such that it was administered in classroom settings with standardized instructions from the first author and three trained local research assistants. At the start of each questionnaire session, the participants were informed by the first author of the purpose of the study, how to complete the questionnaires, and their right to withdraw their participation. To maximize the validity of the self-report data, the maximum number of participants was set at 30 to 40 people per survey occasion in classrooms (which could contain about 80 people). Additionally, the students were instructed to sit apart from one another, to keep their eyes on their own questionnaires, and to not discuss any of the questions with other students while taking the survey. The first author and research assistants were present throughout the administration process to answer possible questions from the participants. No teachers or other university staff were present. Students were assured repeatedly that their responses would be anonymous and would be analyzed in an aggregated manner, with personal information being kept in strict confidentiality. The data collection was approved by the human research ethics committee the University of Hong Kong and by the surveyed universities administration committee.

### Measures

#### Attitudes toward Same-Sex Attraction and Behavior

To assess participants’ attitudes toward same-sex attraction and behavior, we used adapted versions of four items (i.e., “Homosexuality violates social norms,^2^” “Having same-sex sexual impulses is acceptable,” “Having sex with a person of the same sex is acceptable,” and “Same-sex marriage is acceptable”) from an existing Chinese sexual moral values scale (Yi et al., 2007). Respondents answered the items on a Likert scale (1 = *strongly disagree*, 5 = *strongly agree*). The scale had sound construct validity (inter-correlations ranged from 0.30 to 0.50, *p* < 0.01; factor loadings ranged from 0.68 to 0.80) and adequate internal consistency (Cronbach’s α = 0.72). We computed mean scores for the four items (the first item was reversed), with higher scores indicating more positive attitudes.

#### Socio-demographic Correlates

Participants were asked to report their gender (0 *= male*, 1 *= female*), age and number of years in university (heretofore referred to as grade). They also reported their family income (1 = *lower than middle level*, 2 = *middle level*, 3 = *higher than middle level*), their fathers’ and mothers’ education levels [1 *= low* (i.e., illiteracy or primary school), 2 = *middle* (i.e., middle or high school), 3 = *high* (i.e., college or higher)], and whether they grew up in a rural area or an urban area (0 = *rural*, 1 = *urban*). In addition, participants reported whether they had previously received sex education (0 = *no*, 1 = *yes*), and how frequently they spent time online (1 = *every two weeks*, 2 = *every one week*, 3 *= every day*).

### Statistical Analyses

First, frequencies, percentages, means, and standard deviations were computed for all dependent and independent variables (**Table 1**). Second, means and standard deviations were computed for the global attitude measure and for attitude in each item for all participants. Third, a hierarchical regression analysis was performed to explore how the socio-demographic factors correlated with the participants’ reported attitudes. In the first regression step, participants’ mean scores on the attitude questionnaire served as the dependent variable, and gender, grade, family SES, urbanity, sex education experience, and online frequency served as the independent variables. In the second step, interactions between correlates and participants’ genders were entered into the analysis in order to assess potential gender differences in the correlates. All ordinal-level and continuous independent variables were standardized with a z-transformation. Categorical variables were dummy-coded as 0 (i.e., *male, rural*) or 1 (i.e., *female, urban*). All analyses were performed using SPSS for Windows, version 22.0. In the interpretation of the results, the statistical significance was set to *p* < 0.05 (two-tailed)^3^.

## Results

### Attitudes toward Same-Sex Attraction and Behavior

As shown in **Table 2**, the students had negative attitudes toward “having same-sex sexual impulses,” scoring an average of 2.23 (*SD* = 1.22) on a scale of 1 to 5. Students had neutral attitudes toward “homosexuality violates social norms,” scoring an average of 3.07 (*SD* = 1.11). Meanwhile, students had negative attitudes toward same-sex marriage and toward having sex with a person of the same sex, scoring averages of 2.03 (*SD* = 1.11) and 2.51 (*SD* = 0.86), respectively. In general, students scored an average of 2.51 (*SD* = 0.86) across all questions. Male students scored an average of 2.35 (*SD* = 0.81), and female students scored an average of 2.68 (*SD* = 0.86). Thus, generally, Chinese university students reported negative attitudes.

**Table 2:** Means and standard deviations of males, females, and the total sample in each item and the global score.

	Mean ± SD		
Items	All	Male	Female
1. Having same-sex sexual impulses is acceptable	2.23 ± 1.22	2.07 ± 1.22	2.39 ± 1.20
2. Homosexuality violates social norms^a^	3.07 ± 1.11	2.92 ± 1.16	3.22 ± 1.04
3. Same-sex marriage is acceptable	2.72 ± 1.19	2.47 ± 1.16	2.98 ± 1.16
4. Having sex with a person of the same sex is acceptable	2.03 ± 1.11	1.93 ± 1.07	2.13 ± 1.14
5. Global score	2.51 ± 0.86	2.35 ± 0.81	2.68 ± 0.86

*^a^This item was reverse scored, thus, the scores reported for this item are adjusted to reflect more accepting attitudes.*

### Socio-demographic Correlates

As can be seen in **Table 3**, gender, grade, maternal education, urbanity, and frequency of time spent on the Internet emerged as significant main correlates in the regression analysis of university students’ attitudes. Specifically, female students’ attitudes were more positive than males students’ attitudes (β = 0.20, *p* = 0.000). Students in higher grades (i.e., with more years of university education) reported more positive attitudes than students with fewer years in university (β = 0.08, *p* = 0.000). Students whose mothers had higher education levels had more positive attitudes than students whose mothers had lower education levels (β = 0.07, *p* = 0.004). Students who grew up in urban areas held more positive attitudes than those who grew up in rural areas (β = 0.10, *p* = 0.000). Finally, students who spent time on the Internet more frequently held more positive attitudes (β = 0.09, *p* = 0.000). The main effects of paternal education, family income, and sex education experience were not significant.

**Table 3:** Regression analysis results of correlates of Chinese University students’ attitudes toward same-sex attraction and behavior.

	*B*	*SE*	β	*t*	Δ*R*^2^
*Step 1*					0.073***
Gender	0.33	0.03	0.20	10.44***	
Grade	0.07	0.02	0.08	3.90***	
Father education	-0.03	0.02	-0.03	-1.45	
Mother education	0.06	0.02	0.07	2.87**	
Family income	0.02	0.02	0.02	1.09	
Urbanity	0.17	0.04	0.10	4.41***	
Sex education	0.05	0.04	0.02	1.24	
Online frequency	0.07	0.02	0.09	4.38***	
*Step 2*					0.012***
Gender	0.29	0.05	0.17	6.16***	
Grade	0.01	0.02	0.01	0.51	
Father education	-0.05	0.03	-0.06	-1.81	
Mother education	0.03	0.03	0.03	0.88	
Family income	0.02	0.02	0.02	0.63	
Urbanity	0.09	0.06	0.05	1.60	
Sex education	0.08	0.06	0.04	1.43	
Online frequency	0.07	0.02	0.08	3.05**	
Gender × Grade	0.13	0.03	0.10	3.82***	
Gender × Father education	0.04	0.04	0.03	0.98	
Gender × Mother education	0.07	0.04	0.06	1.67	
Gender × Family income	0.01	0.04	0.01	0.13	
Gender × Urbanity	0.14	0.08	0.07	1.81	
Gender × Sex education	-0.04	0.08	-0.02	-0.50	
Gender × Online frequency	0.01	0.03	0.01	0.14	

*p < 0.05; **p < 0.01, ***p < 0.001.

### Gender Interactions

One significant interaction was found between gender and grade (β = 0.10, *p* = 0.000). In order to disentangle this interaction, regression analyses were re-run separately for male and female students, without the gender main effects or the gender interactions. The results of these analyses showed that, for female students, having more years of university education was related to more positive attitudes (β = 0.15, *p* = 0.000). This was not the case for male students (β = 0.02, *p* = 0.608). In other words, for male students, years of university education was not significantly related to attitude. There were no significant interactions between gender and parents’ education, family income, urbanity, sex education experience, or online frequency (**Table 3**).

## Discussion

The aim of the present study was to examine Chinese university students’ attitudes toward same-sex attraction and behavior, as well as several socio-demographic correlates of these attitudes. The study also explored whether gender differences existed in the tendencies and correlates of these attitudes. The results showed that both male and female Chinese university students generally held negative attitudes toward gay and lesbian individuals. This indicates that Chinese young people might still be influenced by traditional values and norms embedded in elements of Confucianism, which guide popular thinking about interpersonal relationships, family responsibilities (e.g., to the family line and filial piety), and sexual conduct (So and Cheung, 2005). These traditions have strong impacts on the broader social, ethical, and moral orientations in Chinese society (Winter et al., 2008). While the present study did not overtly examine participating university students’ adherence to the traditional values of Confucianism, this is most certainly an important issue to examine in future research using Chinese participants. The findings suggest that university-based interventions aiming to increase students’ positive attitudes toward same-sex attraction and behavior might benefit from integrating features of Chinese culture. For example, discussing the cultural underpinnings of negative attitudes and elements of traditional Chinese culture and history that might promote greater acceptance may be useful in increasing the success of existing programs.

Although both male and female Chinese university students reported negative attitudes toward same-sex attraction and behavior, we found that men were significantly more negative in their attitudes than women. This was consistent with both our expectations and the findings of prior studies (Herek, 1988, 2002; Costa and Davies, 2012; Kohut, 2013). Scholars have explained that these findings might relate to men’s more rigid demands for gender role conformity, considering that gender role beliefs are clearly linked to attitudes toward same-sex sexual behavior and relationships (Costa and Davies, 2012). Additionally, men hold higher status and power than women in Chinese society, and it is in their interest to urge conformity to roles that emphasize that advantage (Louie, 2002). One way to accomplish this is through the derogation of individuals who violate these roles (Kite and Whitley, 1996).

As expected, most of the investigated correlates showed significant predictive effects. For both male and female students, higher levels of maternal education, having grown up in an urban area, and more frequent Internet use were linked to more positive attitudes. The findings were largely consistent with previous studies (e.g., Waterman et al., 2001; Teney and Subramanian, 2010; Norton and Herek, 2013). Firstly, whereas higher maternal education levels were linked with more positive attitudes, paternal education level was not a significant predictor of attitude. Considering that mothers are typically the primary caregivers of children in China and spend more time with their children than fathers do (Hou et al., 2005), there is potentially more opportunity for them to transmit their beliefs and attitudes to their offspring. Furthermore, considering that females generally hold more positive attitudes than males and that higher education is usually linked to more tolerant attitudes (McDermott and Blair, 2012; Norton and Herek, 2013), it stands to reason that women with higher education levels might generally hold less prejudices toward sexual minorities. Accordingly, participating students whose mothers had higher levels of education held more positive attitudes. More research could be conducted to further explore the gender differences in the intergenerational transmission (i.e., father–son, mother–son, father–daughter, and mother–daughter) of such values in different cultures.

Regarding the finding that university students from urban areas held more tolerant attitudes than those from rural areas, it might be that urban young people have greater exposure to various social norms and values, including those related to same-sex attraction, behavior, and relationships (Feng et al., 2010). Previous studies have demonstrated that encountering diverse social norms and values and having first-hand social contact with gay and lesbian individuals significantly predict more positive attitudes (Gelbal and Duyan, 2006; Rye and Meaney, 2009).

Although university students in China benefit from more education about same-sex attraction, behavior, and relationships, the results of the present study suggest that male students, students in the early grades of university, students from rural areas, and students whose mothers have attained lower levels education may represent particularly important focus areas of Chinese university educational strategies aiming to increase positive attitudes. This is also in line with research from Western countries, which has found that males, youths with lower levels of education, and youths from rural areas are particularly important targets of psycho-educational efforts (e.g., Waterman et al., 2001).

We also found a relation between a higher frequency of time spent online and more positive attitudes. Western studies have shown that the use of new media, especially the Internet, is significantly associated with more positive attitudes toward sexual minorities (Newman, 2007; Lee and Hicks, 2011). The present study suggests that new media may also be a meaningful correlate of Chinese students’ attitudes, possibly as a substitute to real-life contact. It could be that, in contexts where there is little real-life contact with sexual minorities, students acquire a great deal of information (e.g., more global/Western norms and attitudes, more acceptance of sexual diversity) from the media, including the Internet. These findings are consistent with the findings of other studies indicating that the integration of online resources (e.g., online sex education websites) providing relevant information, education, or training might also help intervention programs foster a more tolerant and open atmosphere toward sexual minorities (Barak and Fisher, 2001; Lou et al., 2006).

In addition, we found one significant gender interaction, in which female students with more years of university education held more positive attitudes, but male students did not show a significant association. This result is similar to the aforementioned finding regarding the positive effect of mothers’ educational attainment, in that females generally hold more positive attitudes than males, and higher education is usually linked to more tolerant attitudes (McDermott and Blair, 2012; Norton and Herek, 2013). Female students might, thus, become increasingly tolerant in their attitudes toward same-sex attraction and behavior as their university education progresses. Future research could utilize longitudinal methods to track the same group of university students in order to examine developmental changes in the attitudes of male and female students over time.

In contrast with our expectations, we found that that having received sex education did not significantly predict attitudes toward same-sex attraction or behavior. This may be because only 22% of our participants reported having received sex education. Moreover, these programs likely varied considerably in their timing, content, and quality, as sex education programs in Chinese high schools and universities are still not uniform in their quantity or quality and usually include only a few selective courses, sporadic lectures, or seminars (Huang et al., 2009). Furthermore, such education is often not compulsory, but instead typically depends on students’ own interest and initiative. An alternative explanation for the lack of a link between sex education and more positive attitudes might be that the topics of same-sex attraction and behavior are often not included in Chinese high school or university courses (Huang et al., 2009). Even when the topic is addressed, it might not be in the context of an open and tolerant atmosphere. Yet, a recent empirical study by Chi et al. (2013) has shown that a university sex education program specifically addressing lesbian, gay, and transgender issues through diverse teaching strategies (e.g., literature readings, lectures, videos, relevant guest speakers, and/or reflective discussions) produced short-term increases in tolerant attitudes among university students. This stresses the importance of including topics related to sexual orientation in sex education programs in order to promote a better understanding and acceptance of lesbian and gay individuals and their experiences.

### Strengths and Limitations

Although the present study has several strengths, including utilizing data collected from a large sample of Chinese university students from multiple universities, there are also several limitations worth noting. First, the instrument used to assess attitudes was a unidimensional questionnaire that could not make finer distinctions between attitudes toward male and female individuals who identify as bisexual, gay (men), or lesbian. As the literature points out, people sometimes differ in their attitudes toward these groups (Steffens and Wagner, 2004); thus, future studies should consider using more nuanced, multidimensional measurements of attitudes toward same-sex attraction and behavior (i.e., by separately examining attitudes toward male and female bisexuals, lesbians, and gay men).

Second, although our data were collected from students from nine different Chinese universities, the results obtained in this study cannot be generalized to all university students, nor to all young people in China, because our student sample was based in only two districts. Future studies should aim to collect nationally representative data on Chinese university students. At the same time, however, specific efforts should be devoted to examining attitudes toward same-sex attraction and behavior among less-educated Chinese youth and young people who are not enrolled in schools or universities at all, such as young immigrant workers who have migrated from villages to cities. Future studies examining attitudes among these groups may provide valuable insights for interventions targeting youths with less education.

Third, while this study examined various socio-demographic correlates, prior research has demonstrated that such factors as religiosity, personal endorsement of Confucian principles, parenting processes, peer relationships, and real-life experiences are also significantly correlated with attitudes toward same-sex attraction and behavior. Future research, thus, should examine more contextual, relational, and personal predictors. Additionally, finer gradients of scales (as opposed to dichotomous or trichotomous response options) might provide more variance in measurement, thus offering a clearer picture of which predictors are the most meaningful.

Fourth, we did not question participating university students about their own sexual orientations. Thus, we do not know the percentage of participants in our study who might have identified as gay, lesbian, bisexual, or questioning, or how this may have been related to their reported attitudes. In future studies, assessments of participants’ own sexual orientation should be added (i.e., through the use of multidimensional scales to assess respondents’ own sexual identities) (Currin et al., 2015).

Finally, the self-report nature of the questionnaires, particularly paper-and-pencil self-administered questionnaire, can be considered a possible limitation, especially when referring to sexuality topic (e.g., sexual orientation). Employing other research methods, such as individually completed online questionnaires, or qualitative methods (e.g., one-one interviews) could be a valuable direction for future studies. These methods could make participants feel more privacy and safety, and therefore more willing to express their true ideas and situation, which may accordingly elicit more open and true response.

Together, the findings of the present study show that various socio-demographic factors that correlate with young people’s attitudes toward same-sex attraction and behavior, as well as gender differences in these attitudes, continue to persist in contemporary China. The current study contributes to the existing body of knowledge on attitudes toward same-sex attraction and behavior among university students in the Chinese context. This has important implications for theory, future research, and intervention practices. Theoretically, the study demonstrates that hegemonic masculinity and traditional Confucian values are still prevalent in contemporary China. Additionally, the study generally confirms female plasticity theory, which suggests that females are more malleable than males. However, a better understanding is needed of the contextual, relational, and personal predictors of Chinese young people’s attitudes and the changes in these attitudes over time. Practically, this study may help in the development of interventions, including elements of formal sexuality education that address same-sex attraction and behavior, tailored to the specific needs of different groups (e.g., males and students from rural areas) in order to more effectively promote Chinese young people’s positive attitudes toward same-sex attraction and behavior.

## Author Contributions

XC and SH contributed to the design of this research. XC performed the statistical analysis and drafted the manuscript. SH supervised the research and statistical analysis and revised the manuscript. The authors both read and approved the final manuscript.

## Conflict of Interest Statement

The authors declare that the research was conducted in the absence of any commercial or financial relationships that could be construed as a potential conflict of interest.

## Footnotes

1 Although the Two-Child Policy replaced the One-Child Policy on January 1, 2016, the One-Child Policy was still in place at the time our data were collected.

2 The item “homosexuality violates social norms” assesses students’ ideas or expectations concerning whether homosexual attraction, same-sex behavior, and same-sex marriage are considered acceptable by the Chinese public. In other words, it asks students whether homosexual attraction, same-sex behavior, and same-sex marriage are the accepted standards of behavior in Chinese society. This item was explained to the survey participants prior to their participation.

3 In this study, we used quantitative method (i.e., regression analysis) to address research questions for several reasons: First, quantitative research method may be the best method to analyze large sample data. The results based on the method may reflect the general idea of one group and may be generalized to a larger population. However, other method such as qualitative research method may not do it. Furthermore, regression analyses are the most suitable when aiming to examine the unique contributions of several variables at the nominal, ordinal, and continuous level. Additionally, regression analyses yield the clearest results with regard to interactions between nominal- and ordinal-level variables. Finally, regression analyses allow the examination of the predictors within one test, as opposed to conducting several *t*-tests (which would both heighten the possibility of false-positive results and increase the overall length of the Results section) or several factorial ANOCOVAs, which would potentially return results for several higher-order interactions (e.g., gender × father education × urbanity) that were not the aim of this study.
